# A Multiscale View of the Mechanisms Underlying Ketamine’s Antidepressant Effects: An Update on Neuronal Calcium Signaling

**DOI:** 10.3389/fnbeh.2021.749180

**Published:** 2021-09-30

**Authors:** Ayako Kawatake-Kuno, Toshiya Murai, Shusaku Uchida

**Affiliations:** ^1^SK Project, Medical Innovation Center, Kyoto University Graduate School of Medicine, Kyoto, Japan; ^2^Department of Psychiatry, Kyoto University Graduate School of Medicine, Kyoto, Japan

**Keywords:** ketamine, antidepressant action, neuroplasticity, epigenetics, gene expression, stress, glutamate receptor, calcium signaling

## Abstract

Major depressive disorder (MDD) is a debilitating disease characterized by depressed mood, loss of interest or pleasure, suicidal ideation, and reduced motivation or hopelessness. Despite considerable research, mechanisms underlying MDD remain poorly understood, and current advances in treatment are far from satisfactory. The antidepressant effect of ketamine is among the most important discoveries in psychiatric research over the last half-century. Neurobiological insights into the ketamine’s effects have shed light on the mechanisms underlying antidepressant efficacy. However, mechanisms underlying the rapid and sustained antidepressant effects of ketamine remain controversial. Elucidating such mechanisms is key to identifying new therapeutic targets and developing therapeutic strategies. Accumulating evidence demonstrates the contribution of the glutamatergic pathway, the major excitatory neurotransmitter system in the central nervous system, in MDD pathophysiology and antidepressant effects. The hypothesis of a connection among the calcium signaling cascade stimulated by the glutamatergic system, neural plasticity, and epigenetic regulation of gene transcription is further supported by its associations with ketamine’s antidepressant effects. This review briefly summarizes the potential mechanisms of ketamine’s effects with a specific focus on glutamatergic signaling from a multiscale perspective, including behavioral, cellular, molecular, and epigenetic aspects, to provide a valuable overview of ketamine’s antidepressant effects.

## Introduction

Major depressive disorder (MDD) is the leading cause of disability worldwide. Despite considerable research, biological mechanisms underlying MDD pathophysiology remain unclear, with significant unmet needs for treatment. Typical antidepressants, including selective serotonin reuptake inhibitors (SSRIs) and serotonin and noradrenaline reuptake inhibitors, increase monoamine concentration in the synaptic cleft, resulting in antidepressant effects (Berton and Nestler, 2006). However, although increased monoamine concentration in the synapse occurs relatively quickly as an acute pharmacological action, recovery from depression takes several weeks to months in clinical practice (Krishnan and Nestler, 2008). Electroconvulsive therapy (ECT) is also an effective treatment for drug-resistant depression, although achieving clinically meaningful or sustained remission with ECT required at least 1 month (Yamasaki et al., 2020). Such substantial time lags are a major concern since patients with depression are at high risk for suicide. Thus, there is an urgent need to develop antidepressants with rapid onset and sustained effectiveness.

Ketamine, a non-competitive glutamate *N*-methyl-D-aspartate receptor (NMDAR) antagonist, has gained considerable interest in the neuropsychiatric field. A single administration of ketamine elicits rapid and sustained antidepressant effects for 1–2 weeks in both humans and animals (Berman et al., 2000; Zarate et al., 2006; Li et al., 2010; Autry et al., 2011). This discovery offered new insight into the investigation of a whole new class of agents beyond the monoamine system to treat depression (Chaki, 2017). *Esketamine*, an enantiomer of (*R,S*)-ketamine, has been approved by the U.S. Food and Drug Administration (USFDA) for treating patients with treatment-resistant depression. Thus, research on pathophysiology and drug discovery for MDD has transitioned from the monoaminergic to the glutamatergic system. Recently, the importance of multiscale neuroscience to study cross-scale interactions at genetic, molecular, cellular, and macroscale levels of brain circuitry, connectivity, and behavior has been emphasized to establish a comprehensive understanding of neuropsychiatric disease (Van Den Heuvel et al., 2019). This mini-review aims to update the current knowledge regarding ketamine effect on the brain, focusing on the glutamatergic signaling pathway from a multiscale perspective at the behavioral, cellular, molecular, and epigenetic levels.

## The Glutamatergic System in Neuroplasticity, Intracellular Signaling, and Gene Expression

Glutamate is the major excitatory neurotransmitter in the brain, and increasing evidence indicates that dysfunction in glutamatergic signaling contributes to MDD pathophysiology (Popoli et al., 2011; Duman and Aghajanian, 2012; Thompson et al., 2015; Duman et al., 2019; Xia et al., 2021). The glutamatergic system is modulated by both ionotropic [NMDARs, α-amino-3-hydroxy-5-methyl-4-isoxazolepropionic acid receptors (AMPARs), and kainate receptors] and metabotropic glutamate receptors (mGluRs). NMDARs are found throughout the central nervous system and contribute to synaptic calcium (Ca^2+^) influx, which is required for activity-dependent synaptic plasticity (Koester and Sakmann, 1998; Reid et al., 2001; Ngo-Anh et al., 2005; Bloodgood and Sabatini, 2007; Carter et al., 2007). NMDAR function is tightly linked to AMPAR, which gates sodium and mediates fast excitatory transmission. Increased AMPAR density in the postsynaptic membrane causes NMDAR-dependent long-term potentiation (LTP) (Huganir and Nicoll, 2013). AMPARs can also have several direct effects on synaptic transmission (i.e., LTP) and intracellular signals without the proper functioning of NMDARs. This NMDAR-independent and AMPAR-dependent intracellular signaling pathway is also hypothesized to underlie ketamine’s antidepressant actions (Zanos et al., 2016; Duman et al., 2019; Wei et al., 2021).

**FIGURE 1:**
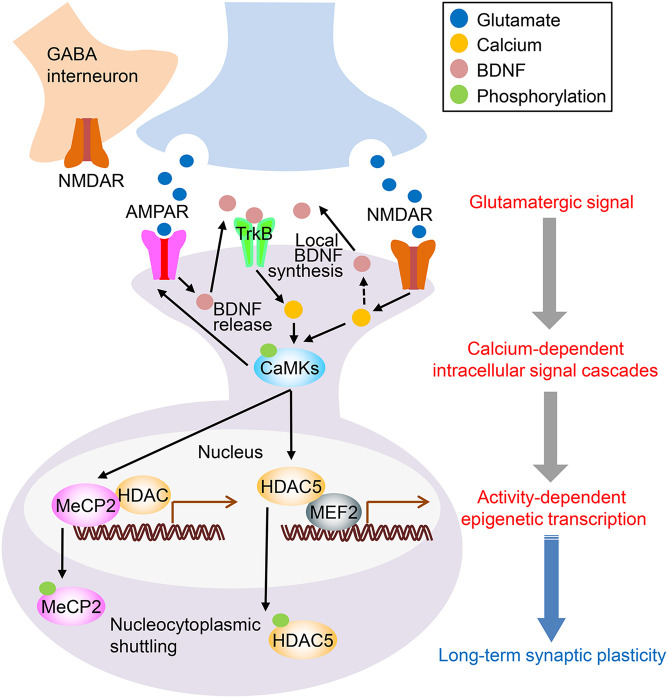
Proposed mechanisms of ketamine’s antidepressant action. The binding of ketamine to *N*-methyl-D-aspartate receptors (NMDARs) on GABAergic interneurons disinhibits glutamatergic neurons, which results in increased synaptic glutamate release. AMPAR activation by glutamate increases brain-derived neurotrophic factor (BDNF) levels. Although the exact source of BDNF is yet to be determined, local release of BDNF is thought to stimulate TrkB receptors. This activation activates intracellular signaling, such as the Ca^2+^ pathway. Another mechanism is the direct inhibition of NMDAR by ketamine. Inhibiting postsynaptic NMDARs reduces eEF2 via the inactivation of CaMK (eEF2 kinase), which leads to enhanced local protein synthesis of BDNF. Increased intracellular Ca^2+^ stimulates CaMKs and their downstream targets, including MeCP2, MEF2, and HDAC5. MeCP2, a transcriptional regulator, binds to methylated CpG sites on the genomic region and interacts with other transcription repressors, including HDACs. CaMKII phosphorylates MeCP2, promotes its nuclear export, and increases activity-dependent transcription. MEF2 recruits HDAC5 and removes activating acetyl groups from histones, which results in a silenced or repressed state of transcription. CaMKII phosphorylates HDAC5, which promotes nuclear export and increases activity-dependent transcription. Ketamine is known to increase the phosphorylation of CaMKII, MeCP2, and HDAC5 (see detail in the main text). Thus, ketamine-mediated enhancement of intracellular Ca^2+^ signaling is linked to epigenetic regulation of transcription, which leads to long-term synaptic plasticity and, consequently, prolonged antidepressant-like effects.

Ca^2+^ influx into the postsynaptic neuron stimulates a signaling-cascade, such as calcium/calmodulin-dependent kinases [CAMKs; e.g., calcium/calmodulin-dependent kinase II (CaMKIIs), eukaryotic elongation factor 2 (eEF2) kinase]. Brain-derived neurotrophic factor (BDNF) and its receptor, neurotrophic receptor tyrosine kinase 2 (TrkB), also plays a key role in synaptic plasticity (Minichiello, 2009). TrkB activation stimulates phospholipase Cγ1 (PLCγ1), which results in CaMK activation (Minichiello, 2009). Calcium-signaling activation further sends its signal toward downstream epigenetic and transcription modulators, such as MEF2, MeCP2, and HDAC5. These pathways modulate gene expression that affects dendritic growth, synaptic development, and neuronal plasticity (Greer and Greenberg, 2008; Graff and Tsai, 2013; Takemoto-Kimura et al., 2017; Uchida and Shumyatsky, 2018a,b; Figure 1). Taken together, calcium-signaling stimulation through NMDARs and/or AMPARs activates multiple downstream nucleocytoplasmic pathways; it induces activity-dependent epigenetic genetic expression, contributing to depression and antidepressant action.

Chronic stress initiates and exacerbates several psychiatric illnesses. Indeed, adverse stressful environments are associated with the pathophysiology of major psychiatric disorders, including mood and anxiety disorders (Mcewen, 2007; Krishnan and Nestler, 2008; Duman and Aghajanian, 2012). There are several evidences demonstrating alterations in the expression and/or function of glutamatergic signaling and its downstream molecules (e.g., NMDARs, AMPARs, CaMKIIs, MEF2, MeCP2, and HDAC5), which is associated with plasticity and behaviors induced by chronic stress, traditional antidepressant drugs, and/or ketamine (Table 1). Moreover, molecular dysregulation associated with glutamatergic system is visible in postmortem brain tissues of patients with MDD (Table 1). Thus, such clinical and preclinical evidences suggest that calcium-signaling is a downstream target of the glutamatergic system in MDD pathophysiology and antidepressant effects.

**TABLE 1:** Example evidence indicates alterations in behavior, glutamatergic signaling, and its downstream pathways regarding depression, chronic stress, and antidepressants: translational and multiscale views.

**Behaviors**		
**Findings**		**References**
Ketamine’s effects on a stress-induced animal model of depression	CUMS-induced increase of immobility in TST were reversed 0.5 and 72 h after ketamine treatment in rats	Sun et al., 2016
	CUS-induced reduction in sucrose preference in SPT was reversed by ketamine 24 h after injection in rats	Li et al., 2011
	CSDS-induced reduction of social interaction was reversed 24 h after (*2R, 6R*)-HNK treatment in mice	Zanos et al., 2016
	CSDS-induced depression-like behaviors were reversed 24 h after (*R*)-ketamine treatment in mice	Yang C. et al., 2018
Ketamine’s effects on pharmacological model of depression	Chronic CORT effects on immobility in TST, open-arm exploration in an elevated plus maze and sucrose preference were reversed 24h after ketamine treatment in mice	Moda-Sava et al., 2019
	Chronic CORT-induced anhedonia in a sucrose preference test was recovered by (*2S, 6S*)-HNK treatment	Zanos et al., 2016
	LPS-induced increase of immobility in FST was reversed by (*R*)- Ketamine, but not (*R*)- HNK, in mice	Yamaguchi et al., 2018
	Chronic CORT-induced anhedonia and increased immobility time in FST were improved by (*2S, 6S*)-HNK, but not (*2R, 6R*)-HNK	Yokoyama et al., 2020
**Neuroplasticity**		
**Findings**		**References**
MDD patients	Postmortem brain of MDD patients showed a lower number of synapses in dlPFC	Kang et al., 2012
	Meta-analysis of structural imaging studies demonstrated that MDD patients have smaller hippocampus volumes	Macqueen and Frodl, 2011
	Meta-analysis of imaging showed the structural and functional decline in dmPFC of MDD patients	Price and Drevets, 2010
Stress-induced animal model of depression	CUS decreases the number and function of spine synapses in the mPFC	Li et al., 2011
	Reduced spine density in the hippocampus and mPFC of mice susceptible to CUMS and CSDS	Abe-Higuchi et al., 2016; Higuchi et al., 2016; Nie et al., 2018; Sakai et al., 2021
	Repeated stress impairs glutamatergic transmission in PFC pyramidal neurons	Yuen et al., 2012
Ketamine’s effect	(S)-ketamine normalized habenula, midline thalamus, and hippocampal connectivity at 48 h in fMRI imaging of stressed rats	Gass et al., 2019
	Ketamine blocks NMDAR spontaneous activity	Autry et al., 2011
	Ketamine treatment restores lost spines by chronic CORT exposure and promote generating functional synapses in mice	Moda-Sava et al., 2019
	Ketamine treatment increases the number and function of spine synapse in rat mPFC	Li et al., 2010
	(*2R,6R*)-HNK increased fEPSC slope in SC-CA1 of rats	Zanos et al., 2016
	(*2S,6S*)-HNK caused no changes in sEPSC frequency or amplitudes in rat CA1 interneurons (but has antidepressant effect)	Chen et al., 2012
**Molecular pathway/Intracellular signaling**		
**Molecules**	**Findings**	**References**
NMDARs	MDD and stress model	
	A postmortem prefrontal cortex showed increased levels of NR1 in MDD	Rodriguez-Munoz et al., 2017
	Reduced GluN2A in prefrontal cortex of MDD	Beneyto and Meador-Woodruff, 2008
	MK801, a NMDAR antagonist, injection reduced immobility in FST	Autry et al., 2011
	CUS-induced reduction in sucrose preference in SPT was reversed by a selective NR2B antagonist, Ro 25-6981, 24 h after injection in rats	Li et al., 2011
	Ketamine	
	Ketamine treatment increases NR1 expression levels in mouse PFC	Liu et al., 2011
	Ketamine and a high dose of (*2R, 6R*)-HNK influences NMDAR-mediated eEF2 phosphorylation	Autry et al., 2011; Suzuki et al., 2017
	(*2R, 6R*)-HNK do not block NMDAR function	Lumsden et al., 2019
AMPARs	MDD and stress model	
	Postmortem cortical tissue from MDD patients showed decreased GluA1 levels	Beneyto et al., 2007
	Reduced GluA1 level in the hippocampus of stress-susceptible mice AMPAR potentiator drives stress resilience, whereas GluA1 inhibition leads to stress susceptibility	Sakai et al., 2021
	Ketamine	
	Ketamine increased the level of GluA1 subunit in the mouse hippocampus	Beurel et al., 2016
	(*2R, 6R*)-HNK increased synaptic GluA1 and GluA2 protein expression in the mouse hippocampus	Zanos et al., 2016
BDNF/TrkB	MDD and stress model	
	Postmortem brain tissues from the hippocampus and prefrontal cortex in suicide subjects showed reduced expression of BDNF and TrkB	Dwivedi et al., 2003
	BDNF levels were lower in the anterior cingulate of postmortem brains of subjects with early life adversity and/or died by suicide	Youssef et al., 2018
	Ketamine	
	CUMS-induced reduction of the expression of BDNF was reversed 0.5 and 72 h after ketamine treatment in rats	Sun et al., 2016
	The deletion of BDNF or TrkB in broad forebrain regions of mice blocks ketamine’s antidepressant effects	Nosyreva et al., 2013, 2014
	Neutralizing a BDNF antibody into the mPFC blocks the behavioral effects of ketamine in FST	Lepack et al., 2014
	(*2R,6R*)-HNK increased BDNF protein levels 24 h after injection in mouse hippocampus	Zanos et al., 2016
	(*2S,6S*)-HNK increased extracellular BDNF levels in the mouse prefrontal cortex	Anderzhanova et al., 2020
CaMKIIs	MDD and stress model	
	A postmortem study showed decreased levels of *CAMK2B* in the anterior cingulate cortex of MDD	Seney et al., 2018
	A postmortem prefrontal cortex study showed decreased levels of *CAMK2A* in MDD	Fuchsova et al., 2015
	A postmortem prefrontal cortex study showed increased levels of *CAMK2A* in MDD	Tochigi et al., 2008
	CaMKIIβ levels in the ventral HPC were lower in mice following CUMS. CaMKIIβ activation reversed depression-like behaviors	Sakai et al., 2021
	Ketamine	
	CaMKIIβ activity is increased at 3 days after ketamine injection	Kim et al., 2021
MeCP2	MDD and stress model	
	p-MeCP2 levels decreased in the hippocampus and prefrontal cortex of suicide victims	Misztak et al., 2020
	MeCP2 complexes determine stress susceptibility and resilience in mice	Uchida et al., 2011
	Ketamine	
	p-MeCP2 is required for ketamine-induced metaplasticity and antidepressant effects	Kim et al., 2021
MEF2C	MDD and stress model	
	MEF2C is one of the candidate risk genes for MDD	Hyde et al., 2016
	Ketamine	
	Ketamine enhances the transcriptional activity of MEF2 in mice hippocampus	Choi et al., 2015
HDAC5	MDD and stress model	
	Increased *HDAC5* level in MDD	Iga et al., 2007; Hobara et al., 2010
	HDAC5 overexpression in the hippocampus disrupts antidepressant-like effect of traditional antidepressant	Tsankova et al., 2006
	HDAC 4/5 inhibitor induces antidepressant-like behavioral effects in mice	Higuchi et al., 2016
	Ketamine	
	Ketamine induces the phosphorylation of HDAC5 at 30 min and 24 h after administration in mice hippocampus	Choi et al., 2015

*CUMS, chronic unpredictable mild stress; CUS, chronic unpredictable stress; CSDS, chronic social defeat stress; CORT, corticosterone; LPS, lipopolysaccharide; HNK, hydroxynorketamine; MDD, major depressive disorder; SSRI, selective serotonin reuptake inhibitor; FST, forced-swimmed test; SPT, sucrose preference test; TST, tail suspension Test; sEPSC, spontaneous excitatory postsynaptic current; fEPSC, field excitatory postsynaptic current; dlPFC, dorsolateral prefrontal cortex; mPFC, medial prefrontal cortex; dmPFC, dorsomedial prefrontal cortex.*

## Mechanisms of Ketamine’s Antidepressant Effects: A Multiscale View

Less than one-third of patients with MDD achieve remission using traditional antidepressant pharmacotherapy (Trivedi et al., 2006). Treatment resistance occurs in up to 30% of patients with MDD (Fava, 2003). However, a single subanesthetic dose of ketamine produces a therapeutic response within a few hours that lasts for several days in patients with depression (Berman et al., 2000; Zarate et al., 2006). Intravenous infusion of ketamine results in clinical response and remission in 70 and 30% of treatment-resistant patients with MDD, respectively (Zarate et al., 2006). Additionally, Ketamine reduces suicidal ideation (Krystal et al., 2013). In 2020, *esketamine* was approved by the USFDA for treating depressive symptoms in adults with MDD having acute suicidal ideation or behavior.

Ketamine elicits robust unwanted side effects, including prepulse-inhibition deficits, cognitive deficits, and schizophrenia-like psychotic symptoms in humans (Lahti et al., 1995; Chan et al., 2013; Giorgetti et al., 2015). Recent preclinical data indicate that ketamine’s enantiomer (*R*)-ketamine (Hashimoto, 2019; Wei et al., 2021) and its metabolites (*2R, 6R*)-hydroxynorketamine (HNK) (Zanos et al., 2016) exert antidepressant effects with fewer adverse effects than do ketamine or (*S*)-ketamine. Since potential mechanisms underlying the rapid antidepressant actions of ketamine and its metabolites have been reviewed elsewhere (Fukumoto et al., 2017; Yang C. et al., 2018; Duman et al., 2019; Krystal et al., 2019; Sial et al., 2020; Highland et al., 2021; Shinohara et al., 2021; Wei et al., 2021; Xia et al., 2021), we review the recent progress in deciphering mechanisms underlying ketamine’s sustained antidepressant effects, with a particular focus on the role of calcium signaling from a multiscale perspective.

### Behavioral Effects of Ketamine

Several animal studies have demonstrated antidepressant-like responses to ketamine. A single intraperitoneal injection of ketamine or its metabolites produces rapid (30 min–1 h) and long-lasting (24 h–7 days) antidepressant effects (Autry et al., 2011; Koike et al., 2011; Zhou et al., 2014; Sun et al., 2016; Zanos et al., 2016; Yang C. et al., 2018; Kim et al., 2021). Moreover, such ketamine antidepressant effects have been observed in not only naïve, non-stressed animals but also in animals subjected to adverse stressful life events. Animals exposed to chronic stress show despair-like behavior, anhedonia, anxiety, and/or social avoidance, whereas a single injection of ketamine or its metabolites rapidly reverses these deleterious effects and exerts long-term effects (Li et al., 2011; Zanos et al., 2016; Duman et al., 2019; Wei et al., 2021).

### Neurobiological Effects of Ketamine

Neuroimaging studies have shown structural and functional alterations in the hippocampus and dorsomedial prefrontal cortex (dmPFC) of patients with MDD (Price and Drevets, 2010; Macqueen and Frodl, 2011). Human functional magnetic resonance imaging (MRI) studies have demonstrated that a single dose of ketamine ameliorates reductions in functional connectivity in the prefrontal cortex (PFC), which is associated with the alleviation of depressive symptoms (Abdallah et al., 2017). Interestingly, a recent MRI study in animals demonstrated short- and long-term effects of ketamine on distinct brain circuitry. Gass et al. (2019) found in an animal model of depression that ketamine causes a rapid response in the amygdala, anterodorsal hippocampus, and ventral pallidum, which are related to cognitive, sensory, emotional, and reward functions. However, 48 h after administration, ketamine showed a long-term normalization of the habenula, midline thalamus, and hippocampal connectivity. They mediate cognitive flexibility for processing contextual information, distinguish contextual cues in safe versus threatening situations, and modulate fear and emotional responses in non-threatening environments (Gass et al., 2019).

There is increasing evidence suggesting altered neuronal and structural plasticity in animal models of depression as well as in patients with MDD (Duman and Aghajanian, 2012; Kang et al., 2012; Abe-Higuchi et al., 2016; Higuchi et al., 2016; Nie et al., 2018; Uchida et al., 2018; Sakai et al., 2021). Ketamine rapidly increases the number and function of spine synapses. Furthermore, Li et al. found that ketamine increases the number and function of spine synapses in the medial PFC (mPFC) and rapidly reverses synaptic abnormalities caused by chronic stress exposure (Li et al., 2010). Although this evidence suggests an association between ketamine-induced spinogenesis and antidepressant-like behavior, the causal relationship is unclear. However, a recent report by Moda-Sava et al. has addressed this issue. They used a photoactivable proof to selectively reverse ketamine effects on spine formation in the PFC. They found that newly formed spines are necessary for and play a specific role in the sustained antidepressant-like behavior induced by ketamine treatment (Moda-Sava et al., 2019).

### Ketamine-Induced Synaptic Plasticity

Brain-derived neurotrophic factor and its receptor TrkB play key roles in synaptic plasticity, stress, and depression (Duman and Monteggia, 2006; Minichiello, 2009; Castren and Monteggia, 2021). A recent report discovered that several antidepressants, including fluoxetine, imipramine, and ketamine, directly bind to TrkB, facilitating BDNF action and plasticity (Casarotto et al., 2021). In addition, increased BDNF-TrkB signaling in rodent frontocortical/hippocampal circuits has been observed following acute treatment with ketamine (Li et al., 2010; Autry et al., 2011).

Clinical evidence suggests that repeated ketamine administration allows cumulative and sustained antidepressant effects and that it is more effective than a single injection in patients with MDD (Aan Het Rot et al., 2010; Murrough et al., 2013; Phillips et al., 2019). The threshold and sensitivity of the persistent increase and decrease of synaptic strength are subject to activity-dependent regulation. This type of plasticity, called “metaplasticity,” is important for stabilizing synaptic strength and preventing LTP saturation and long-term depression, leading to homeostatic alternations of synaptic activation (Bienenstock et al., 1982; Turrigiano et al., 1998; Kavalali and Monteggia, 2020). Notably, a preclinical study suggested that ketamine administration elicits metaplastic effects on LTP modulation and potentially other processes for long term. Kim et al. (2021) reported that, by using slice recordings of the Schaffer collateral-CA1 pathway in the hippocampus, ketamine induces AMPAR-mediated synaptic potentiation. Interestingly, this effect was more than two-fold higher in brain slices of mice that had received ketamine 7 days earlier, suggesting a priming effect of ketamine treatment such that subsequent ketamine augments synaptic potentiation. Further experiments to understand the mechanisms of this metaplasticity will provide critical insight into mechanisms underlying ketamine’s potent and prolonged antidepressant effects.

### Ketamine-Induced Ca^2+^ Signaling Cascades

*N*-methyl-D-aspartate receptors activate eEF2 via CaMKs (eEF2 kinases) and depress BDNF levels (Scheetz et al., 2000). Ketamine-induced suppression of postsynaptic NMDARs deactivates eEF2 kinase, leading to reduced eEF2 phosphorylation and increased translation of BDNF in the hippocampus (Autry et al., 2011; Suzuki and Monteggia, 2020). This signaling pathway then potentiates synaptic AMPAR responses through the insertion of GluA1/2 subunits (Autry et al., 2011). In contrast, ketamine’s metabolite (*2R, 6R*)-HNK has NMDAR inhibition-independent antidepressant actions (Zanos et al., 2016; Lumsden et al., 2019), whereas other reports have shown that NMDAR inhibition at a high dose of (*2R, 6R*)-HNK triggers intracellular signaling via eEF2 (Suzuki et al., 2017).

A transient burst of glutamate via NMDAR blockade on GABAergic interneurons by ketamine activates postsynaptic AMPARs in excitatory neurons. This activation induces depolarization and activation of NMDARs that trigger Ca^2+^ influx, releasing BDNF (Krystal et al., 2019). Local release of BDNF is thought to activate TrkB on the postsynaptic membrane, stimulating the ERK and PI3K-Akt signaling pathways and mammalian target of rapamycin complex 1 (mTORC1) phosphorylation to promote synapse formation by stimulating synaptic proteins, such as GluA1 and PSD-95, which are required for synaptic plasticity (Cavalleri et al., 2018). Recently, mTORC1 effectors 4E-BP2 and 4-EB2 in excitatory or inhibitory neurons underlie behavioral and neurobiological responses to ketamine (Aguilar-Valles et al., 2021). Ketamine-induced activation of TrkB increases GSK-3β phosphorylation via the ERK signaling pathway, decreasing PSD-95 phosphorylation and internalizing the AMPA GluA1 subunit, which upregulates signaling through the GluA1 to promote synapse formation (Liu et al., 2013; Beurel et al., 2016). Ketamine-dependent changes in dendritic arborization and soma size are abolished by AMPAR antagonists or mTOR complex/signaling inhibitors (Cavalleri et al., 2018). Intracellular molecular signaling cascades stimulated by the glutamatergic pathway may be associated with ketamine-induced structural and synaptic plasticity and its antidepressant effects.

As mentioned earlier, CaMKIIs are major downstream target for the glutamatergic pathway and might be involved in stress and depression. TrkB activation stimulates phospholipase Cγ1 (PLCγ1) and also results in the activation of CaMKs (Minichiello, 2009). Activated CaMKIIs further stimulate MeCP2 phosphorylation (Zhou et al., 2006), allowing the transcription of downstream target genes. A recent study showed that MeCP2 phosphorylation at S421 (p-MeCP2) is essential for the expression of metaplasticity and the sustained, but not acute, antidepressant effects of ketamine (Kim et al., 2021). Hippocampal BDNF protein levels were shown to increase rapidly 30 min after ketamine administration but returned to baseline 3 days after injection. In contrast, hippocampal p-MeCP2 levels increased 3 and 7 days, but not 30 min, after ketamine injection. CaMKIIβ were elevated at 3 days after ketamine injection but returned to baseline at 7 days. These findings indicate that CaMKIIβ plays a role in the intermediary process between BDNF activation and MeCP2 phosphorylation required for the sustained antidepressant effects of ketamine. This hypothesis is also supported, at least in part, by a recent finding that hippocampal CaMKIIβ is downregulated in chronic stress-susceptible mice and that short-term (within 4 days) CaMKIIβ activation ameliorates depression-like behaviors (Sakai et al., 2021).

### Epigenetic Regulation of Gene Transcription by Ketamine

The interplay between genetic and environmental factors underlies depression pathophysiology, and epigenetic mechanisms might contribute to these interactions (Nestler et al., 2016; Uchida et al., 2018; Kawatake-Kuno et al., 2021). Although accumulating evidence demonstrated altered epigenetic functioning in animal models of depression and postpartum MDD-patient brains, few studies have used ketamine-induced transcriptome and epigenome analyses to characterize ketamine’s antidepressant effects. Genome-wide transcriptome and epigenome mapping offer a template for several strategies to identify novel drug targets in unbiased ways to develop more effective treatments for MDD (Bagot et al., 2017). Here we summarize how ketamine-induced activation of Ca^2+^ signal influences epigenetic regulation of gene transcription.

MeCP2, MEF2, and HDAC5 functions are regulated by Ca^2+^ signaling and are associated with stress and depression (Table 1). As mentioned above, p-MeCP2 is necessary for sustained antidepressant response to ketamine (Kim et al., 2021). MeCP2 is a methylated cytosine reader that impacts chromatin organization with any change in DNA methylation. A previous report showed that chronic stress differentially modulates MeCP2 activity in stress-resilient and -susceptible mice and subsequent epigenetic gene transcription (Uchida et al., 2011). Thus, ketamine-induced enhancement of p-MeCP2 may be associated with the formation of chromatin-remodeling complexes on target genes and, thus, transcription regulation. HDAC5 is a histone deacetylase, and its phosphorylation by CaMKs is associated with transcription repression (Mckinsey et al., 2000). Hippocampal HDAC5 is associated with behavioral response to chronic stress and traditional antidepressants (e.g., imipramine and SSRIs) (Tsankova et al., 2006; Higuchi et al., 2016). A recent study suggested that ketamine rapidly induces HDAC5 phosphorylation and nuclear export through CaMKII-dependent pathways, which leads to enhanced MEF2 transcription that regulates neuronal structural and functional plasticity (Choi et al., 2015). Correspondingly, HDAC5 knockdown occludes the actions of ketamine. Moreover, MeCP2 is considered as a master regulator of metaplasticity (Chen et al., 2012). Ca^2+^-signal-mediated modulation of MeCP2, HDAC5, and MEF2 functions may be involved in the sustained antidepressant response of ketamine through epigenetic transcription.

## Conclusion

This mini-review highlights that the glutamatergic pathway is associated with behavioral, neuroplastic, neurobiological, molecular, and epigenetic effects of ketamine, focusing on Ca^2+^ signaling wherein its dysfunction is involved in depression pathophysiology according to both clinical and animal studies. Such (reverse) translational implications for bridging the research gap between human depression and animal models will provide a better understanding of how ketamine affects and modulates depression pathophysiology and ultimately contribute to the clinical application of ketamine or the development of related compounds for wide range of psychiatric disorders. Glutamatergic transmission and monoaminergic systems induce rapid biological changes that induce fast antidepressant effects. In contrast, ketamine’s sustained antidepressant actions are likely mediated by intracellular Ca^2+^ signaling cascades that affect neurobiological processes, including dendritic spine formation, epigenetic modifications, and long-term synaptic plasticity, and consequently, maintain physiological functioning.

In this mini-review, we particularly focused on the hippocampus and prefrontal cortex, key brain regions associated with MDD pathophysiology and ketamine’s antidepressant effect. However, other brain regions were suggested to also be involved in these processes, such as the lateral habenula. Emerging evidence from preclinical and clinical studies identified an important role of the lateral habenula in depression and ketamine’s antidepressant effect through a glutamatergic pathway (Li et al., 2013; Cui et al., 2018a,b, 2019; Yang et al., 2018; Hu, 2019, Hu et al., 2020). In addition, dynamic molecular changes were observed in the nucleus accumbens of animal models of depression and ketamine-treated animals (Bagot et al., 2017). Thus, future studies are warranted to clarify how ketamine impacts neuronal circuit activity and identify underlying molecular and epigenetic mechanisms.

In summary, ketamine has great potential in the development of groundbreaking neuropsychiatric therapies. Our current understanding of depression pathophysiology and ketamine’s action suggests that diverse drug actions converge around Ca^2+^-signaling-mediated neural plasticity. However, ketamine plays diverse roles in the glutamatergic pathway and other neurotransmitter systems, neurogenesis, inflammation, and even body–brain crosstalk. Furthermore, several studies have suggested the distinct roles of ketamine enantiomers ([*S*]-ketamine and [*R*]-ketamine) and their metabolites ([*2R,6R*]-HNK and [*2S,6S*]-HNK) in plasticity and behavior (Zanos et al., 2016; Yamaguchi et al., 2018; Hashimoto, 2019; Lumsden et al., 2019; Yokoyama et al., 2020; Highland et al., 2021; Wei et al., 2021). Thus, mechanisms underlying ketamine’s actions remain controversial. Moreover, ketamine effects at the mesoscale of neural architecture and macroscale of neural connectivity, cognition, and behavior are poorly understood. Further investigations at both the multiscale and multisystem levels are necessary to comprehensively understand mechanisms underlying ketamine’s antidepressant effects and develop novel drugs for treating MDD.

## Author Contributions

All authors listed have made a substantial, direct and intellectual contribution to the work, and approved it for publication.

## Conflict of Interest

The authors declare that the research was conducted in the absence of any commercial or financial relationships that could be construed as a potential conflict of interest.

## Publisher’s Note

All claims expressed in this article are solely those of the authors and do not necessarily represent those of their affiliated organizations, or those of the publisher, the editors and the reviewers. Any product that may be evaluated in this article, or claim that may be made by its manufacturer, is not guaranteed or endorsed by the publisher.
